# Supplementary material for: Flexor tendon degeneration affects short-term outcomes of open trigger digit release
Source: PLoS One. 2023 May 30;18(5):e0286301. doi: 10.1371/journal.pone.0286301 (PMC10228765; doi:10.1371/journal.pone.0286301)
Supplement: Attachment — Submitted filename: PONE-D-22-30862_review.docx [file pone.0286301.s002.docx]

Title: Flexor tendon degeneration affects short-term outcomes of open trigger finger release (PONE-D-22-30862)

**Reviewer Comments**

This is a prospective study evaluating the correlation between flexor tendon degeneration and the outcome of open A1 pulley release for trigger digits treatment.

I have some questions and comments regarding this study, as listed below:

In general:

- To be more specific, trigger digits refer to trigger thumb and trigger finger. Trigger fingers include the index, middle, ring, and small fingers. You can replace the term of trigger finger to trigger digits, or include the separate data of trigger fingers and trigger thumb.

Introduction:

- Line 49: The proximal part of the A2 pulley could also be a cause of trigger finger. (see: J Hand Surg Eur Vol, 2007, 32:521)

Methods:

- Line 81: It would be better to add the details of the grading of trigger finger by Green et al. in the text or a table.
- Line 85: The position setting of the JAMAR® Hand Grip Dynamometer used for testing (first, second, third...) should be mentioned. Different position settings will yield different grip strength.
- Line 94, 95: Did any of the patients undergo partial proximal A2 release? This should be clarified as the proximal A2 pulley release could be a key for some patients to achieve a complete triggering release.

Results:

- How about the correlation of tendon degeneration with the steroid injection? In addition, value to this study would be added if the outcomes of the patients with and without steroid injection could be presented and compared.

Discussion:

- Line 249, 250: “Initially, trigger finger may present as a painless clicking digit.” This is not completely true since many patients initially present with a painful digit. In addition, in the Green classification of trigger finger, grade 1 already includes “pain.”
- You included 12 digits of grade 4, indicating the trigger status in the fixed flexion contracture preoperatively. This group of patients is unique, not only their preoperative flexion contracture, but also the postoperative high chance of residual flexion contracture. You can summarize the macroscopic finding of this group of patients which is an important information. However, the functional influence by this group of patients would cause study bias which you also need to clarify.

Dear Editor-in-Chief,

Thank you for inviting me to review this manuscript.

This is a prospective study evaluating the correlation between flexor tendon degeneration and the outcome of open A1 pulley release for trigger digits treatment. The designs of the study and the manuscript are acceptable. The results are also of help for trigger digits treatment. In my opinion, the manuscript would be acceptable after some revision.

Sincerely,

Jung-Pan Wang
