# Supplementary material for: Flexor tendon degeneration affects short-term outcomes of open trigger digit release
Source: PLoS One. 2023 May 30;18(5):e0286301. doi: 10.1371/journal.pone.0286301 (PMC10228765; doi:10.1371/journal.pone.0286301)
Supplement: Attachment — Submitted filename: Updated Response to reviewers PLOS1 FDS degen.docx [file pone.0286301.s003.docx]

**RESPONSE TO REVIEWERS**

5. Review Comments to the Author

Please use the space provided to explain your answers to the questions above. You may also include additional comments for the author, including concerns about dual publication, research ethics, or publication ethics. (Please upload your review as an attachment if it exceeds 20,000 characters)

Reviewer #1: The authors present an ambitious prospective study evaluating the effect of tendon degeneration on postoperative outcome following open trigger finger release.
The manuscript requires an English revision.
English revision had been done.

The introduction is short and on point.
In the methods section, please specify at what age you considered patients to be adults.

More than 18 years old and above. This had been included. Line 80-81.

I would also suggest to add the specifics of the grading system by Green that you used, as not all readers will be familiar with it.

Green’s classification had been added. Line 101.

Who performed the preoperative assessment and when was it performed? Orthopaedics registrars assisted by a research assistant performed the assessments prior to surgery at minor operating theatre. This had been added in manuscript. Line 86.

Did you use the English version or a translated version of the DASH?
English version.

When analysing the pictures, did the two surgeons do it together? Or separately? If separately, how did you handle the cases where the surgeons disagreed?
The analysis was done by 2 surgeons separately. Training was done for both surgeons prior to assessment so they could identify and quantify the severity of tendon degeneration features (Likert scale 5) in a reliable and consistent manner. In this study, the scores obtained were averaged. Line 132-135.

How did you define comorbidity?
Medical conditions diagnosed by a medical professional and patients are on treatment/ follow ups.

The authors have included several digits from the same patients, and this is a recurrent problem in hand surgical research - how to handle multiple digits from the same patients. Perhaps a small elaboration on this is the discussion would add to the manuscript - for example, it is more likely that a patient with diabetes is represented by more than one finger in the data set than a patient who is otherwise healthy.

In this study, the trigger digit is an idiopathic condition which affects all patients with varying degree of severity and number of digits involved. Thus, analysis was done based on number of digits involved.

I would also suggest to add a regression analysis, to be able to adjust for confounding factors. Is the seen effect of tendon degeneration on ROM and DASH a true effect, or does age and comorbidity play a role?

The objective of this study is to investigate the association between flexor tendon degeneration and outcome of open trigger digit release while looking at the improvement of pain, ROM MCPJ/ PIPJ, pinch/ grip strength and DASH score pre- and post-surgery. The objective of this study does not require regression analysis. From previous literatures, tendinopathies may affect the outcome of trigger release. Age and comorbidity that does not involve musculoskeletal system, do not affect pain, ROM and DASH score. Comorbidity which involves musculoskeletal system like neurological disorder and inflammatory arthritis were excluded from this study.

In the results section, I was very surprised to see that patients had on average 34 visits to the physio before surgery, and few patients had recieved corticosteroid injections beforehand. This differs quite substantially from my practice. Is it standard in your practice that patients have such an extensive treatment by physios before surgery?

Yes. In our centre, many patients refused surgery even after counselling of its benefits. They preferred physiotherapy and were seen weekly. From our data, there were 2 patients who had 50 sessions and 8 patients who had about 40 sessions.

I am also curious of how you reason with corticosteroids, would a previous injection affect the appearance of the tendon?
We identified 26 trigger digits which had steroid injections. Correlation between number of steroid injections with severity of tendon degeneration was done and included in manuscript. Line 196-202 (results) and line 283-290 (discussion). Table 4 had been revised too.

In the discussion, it is mentioned that sonographic assessment was made, however this is not specified in the methods- or is line 210-211 only referring to previous literature?
Yes. From previous literatures, there were 2 methods of describing flexor tendon degenerations; sonographically and histologically. We did not perform these 2 methods. We described the macroscopic features of tendon degeneration via intraoperative imagings.

Figure 4 is difficult to read and requires a higher pixel solution.

Higher pixel of Figure 4 had been included.

Reviewer #2: This is a prospective study evaluating the correlation between flexor tendon degeneration and the outcome of open A1 pulley release for trigger digits treatment.
I have some questions and comments regarding this study, as listed below:

In general:
- To be more specific, trigger digits refer to trigger thumb and trigger finger. Trigger fingers include the index, middle, ring, and small fingers. You can replace the term of trigger finger to trigger digits, or include the separate data of trigger fingers and trigger thumb.
The term trigger finger had been replaced with trigger digit.

Introduction:
- Line 49: The proximal part of the A2 pulley could also be a cause of trigger finger. (see: J Hand Surg Eur Vol, 2007, 32:521)
This information had been added and the reference is included. Line 52-53.

Methods:
- Line 81: It would be better to add the details of the grading of trigger finger by Green et al. in the text or a table.

A table had been added. Line 101.

- Line 85: The position setting of the JAMAR® Hand Grip Dynamometer used for testing (first, second, third...) should be mentioned. Different position settings will yield different grip strength.

Description of positioning of JAMAR® Hand Grip Dynamometer had been included in manuscript. Line 94-100.

- Line 94, 95: Did any of the patients undergo partial proximal A2 release? This should be clarified as the proximal A2 pulley release could be a key for some patients to achieve a complete triggering release.

None of our patients had proximal A2 pulley release because the triggering had completely resolved after A1 pulley release. This had been included in ‘Intraoperative Examination’. Line 121-122.

Results:
- How about the correlation of tendon degeneration with the steroid injection? In addition, value to this study would be added if the outcomes of the patients with and without steroid injection could be presented and compared.

We identified 26 trigger digits which had steroid injections. Correlation between number of steroid injections with severity of tendon degeneration was done and included in manuscript. Line 196-202 (results) and line 283-290 (discussion). Table 4 had been revised too.

Discussion:
- Line 249, 250: “Initially, trigger finger may present as a painless clicking digit.” This is not completely true since many patients initially present with a painful digit. In addition, in the Green classification of trigger finger, grade 1 already includes “pain.”

We had revised the statement. Line 275-277.

- You included 12 digits of grade 4, indicating the trigger status in the fixed flexion contracture preoperatively. This group of patients is unique, not only their preoperative flexion contracture, but also the postoperative high chance of residual flexion contracture. You can summarize the macroscopic finding of this group of patients which is an important information. However, the functional influence by this group of patients would cause study bias which you also need to clarify.

We agree that grade IV trigger digit group is unique. We speculated that the flexor tendon degeneration would be severe in this group. However, there was no correlation between severity of flexor tendon degeneration with severity of trigger finger (grading). Thus, we felt there is no need for subgroup analysis.
