# Supplementary material for: Flexor tendon degeneration affects short-term outcomes of open trigger digit release
Source: PLoS One. 2023 May 30;18(5):e0286301. doi: 10.1371/journal.pone.0286301 (PMC10228765; doi:10.1371/journal.pone.0286301)
Supplement: Attachment — Submitted filename: Response to Reviewers.docx [file pone.0286301.s004.docx]

**RESPONSE TO REVIEWERS**

Reviewer #1: Thank you for the revised manuscript. I only have a few small comments as follows:

Please add to the manuscript that you used the English version of the DASH questionnaire.

This had been added at Line 92 and 149.

Please add your definition of comorbidity to the manuscript.

This had been added at Line 184.

Interesting results with the steroid injections. I would suggest to change the heading in Table 4 from "Number of steroid injections" to "previous steroid injection".

This had been changed in Table 4.
